# Effect of preparation design and endodontic access on fracture resistance of zirconia overlays in mandibular molars: An in vitro study

**DOI:** 10.1111/jopr.13865

**Published:** 2024-05-12

**Authors:** Carlos A. Jurado, Kelvin I. Afrashtehfar, Manuel Robles, Razan S. Alaqeely, Hussain D. Alsayed, Terry J. Lindquist, Abdulaziz Alhotan

**Affiliations:** ^1^ Operative Dentistry Division Department of General Dentistry University of Tennessee Health Science Center College of Dentistry Memphis Tennessee USA; ^2^ Department of Reconstructive Dentistry and Gerodontology (RekGero) School of Dental Medicine, University of Bern Bern Switzerland; ^3^ Evidence‐Based Practice Unit (EBPU) Clinical Sciences Department College of Dentistry Ajman University Ajman City UAE; ^4^ Prosthodontics Private Practice, Dental Clinics Abu Dhabi UAE; ^5^ Artificial Intelligence Research Center (AIRC) Ajman University Dubai UAE; ^6^ Department of Restorative Dentistry Universidad del Valle De Mexico Hermosillo Sonora Mexico; ^7^ Department of Periodontics, College of Dentistry King Saud University Riyadh Saudi Arabia; ^8^ Department of Prosthetic Dental Sciences, College of Dentistry King Saudi University Riyadh Saudi Arabia; ^9^ Department of Prosthodontics The University of Iowa College of Dentistry and Dental Clinics Iowa City Iowa USA; ^10^ Dental Health Department, College of Applied Medical Sciences King Saud University Riyadh Saudi Arabia

**Keywords:** dental prosthesis design, dental restoration failure, dental stress analysis, endodontic access, endodontically‐treated teeth, finish line, fracture resistance, fracture toughness, occlusal veneers, zirconium oxide

## Abstract

**Purpose:**

To evaluate the fracture resistance of zirconia overlays, considering various preparation designs and the presence of endodontic access.

**Materials and Methods:**

Ninety translucent zirconia (5Y‐PSZ) overlay restorations were divided into six groups (*n* = 15/group) based on different preparation designs, with and without endodontic access: chamfer margin 4 mm above the gingival level without (group 1) and with endodontic access (group 2); margin 2 mm above the gingival level without (group 3) and with endodontic access (group 4); overlay with no chamfer margin without (group 5) and with endodontic access (group 6). Restorations were bonded to mandibular first molar resin dies, and the groups with endodontic access were sealed with flowable resin composite. All restorations underwent 100,000 cycles of thermal cycling between 5°C and 55°C, followed by loading until fracture. Maximum load and fracture resistance were recorded. ANOVA with Tukey post‐hoc tests were used for statistical comparison (*α* < 0.05).

**Results:**

Fracture resistance significantly varied among overlay designs with and without endodontic access (*p* < 0.001), except for the no‐margin overlays (groups 5 and 6). Overlays with a 2 mm margin above the gingival margin with endodontic access (group 4) exhibited significantly higher fracture resistance compared to both the 4‐mm supragingival (group 2) and no‐margin (group 6) designs, even when compared to their respective intact groups (groups 1 and 5). There were no significant differences between the no‐margin and 4‐mm supragingival overlays.

**Conclusion:**

The more extensive zirconia overlay for mandibular molars is the first choice since the 2 mm margin above the gingival level design withstood considerable loads even after undergoing endodontic access. A no‐margin overlay is preferred over the 4‐mm supragingival design as it preserves more tooth structure and there was no outcome difference, irrespective of endodontic access. Caution is warranted in interpreting these findings due to the in vitro nature of the study.

Conservative tooth preparations are designed to minimize the removal of tooth structure while preserving as much tooth integrity as possible.^1^ This approach ensures sufficient space for the required restorative material. In contrast, traditional indirect full‐coverage single‐unit restorations (i.e., single crowns) entail a less conservative approach as studies indicate they demand the removal of 24%–70% of the tooth structure, which can compromise its longevity.^2^ Conservative tooth preparations help reduce trauma to the pulpal tissue^3^ and prevent subgingival finish lines that could irritate the periodontal tissues.^4^ Partial coverage crowns, including overlay restorations, have been proposed as an alternative to traditional single crowns (SCs) for managing occlusal wear and restoring masticatory function.^5^, ^6^ Overlay restorations, partial restorations covering the occlusal surface, are referred to with various terms, including occlusal veneers, table tops, or partial restorations in the literature.^7^ These restorations can be advantageous due to their preservation of the tooth structure.^8^, ^9^

In the last decade, computer‐aided design and computer‐aided manufacturing (CAD‐CAM) technology has witnessed significant expansion within restorative dentistry.^10^, ^11^ Clinicians can perform a spectrum of procedures, both simple and complex, using fully digital workflows ranging from single‐tooth to full‐mouth scenarios.^12^, ^13^ These workflows have demonstrated their potential to reduce human error, improving speed, accuracy, and predictability of restoration fabrication.^14^, ^15^ Furthermore, chairside CAD‐CAM technology allows clinicians to produce diverse ceramic restorations without requiring a dental technician.^16^ This technology also facilitates the fabrication of novel partial restorations such as overlays. Partial restorations, particularly CAD‐CAM ceramic overlays, have garnered attention for their efficacy, as evidenced by multiple case studies^17^, ^18^, ^19^ and supported by a recent systematic review and meta‐analysis suggesting positive short‐term results.^20^

Endodontic therapy is a common procedure performed by clinicians, with an estimated 15 million root canal treatments performed annually in the United States alone.^21^ Previous studies have shown that complications can occur after completion of a crown, with 3% requiring subsequent endodontic therapy.^22^ Access through the prosthesis is sometimes used to avoid the need to refabricate an SC or fixed dental prosthesis. A clinical retrospective study evaluated almost 50,000 SCs over 10 years and found that over 2.6% required endodontic treatment, highlighting the potential for complications.^23^ While some case reports have described successful endodontic treatment through SCs,^24^, ^25^ limited data evaluate the impact of endodontic access on the fracture resistance of overlay restorations. Thus, it is important to explore how endodontic access might affect the durability of overlay restorations, addressing a gap in the current body of knowledge.

Therefore, this study aimed to evaluate the fracture resistance of overlay restorations with different designs, with and without endodontic access. The first null hypothesis was that there would be no difference in fracture resistance of zirconia overlays with three different preparation designs (i.e., no margin, 2 mm, and 4 mm supragingival finish line). The second hypothesis was that there would be no difference in fracture resistance between zirconia overlays with varying preparation designs, regardless of the presence or absence of endodontic access.

## MATERIALS AND METHODS

Three typodont mandibular right first molars (1560 Dentoform, Columbia Dentiform, Lancaster, PA, USA) were prepared for zirconia overlay restorations. The preparations involved a 1.0 mm occlusal reduction with a chamfer finish line located at 2 and 4 mm above the gingival level and without a finish line. The three typodont teeth were scanned (Aoralscan, Shinning 3D Dental, Hangzhou, China) and the restorations were digitally designed (DentalCAD 3.1 Rijeka, Exocad, Darmstadt, Germany) following the tooth preparations. Ninety restorations were milled out (DWX 52D, Roland DGA, Irvine, CA, USA) of translucent zirconia 5Y‐PSZ (Katana, Noritake Kuraray) and divided into six groups (*n* = 15/group). The restorations were fabricated with 60 µm cement space as recommended in the literature.^26^, ^27^, ^28^, ^29^, ^30^ CAD‐CAM technology ensured each restoration adhered to high‐quality standards, yielding uniformly quality specimens with no exclusions needed. Post‐fabrication checks for flaws or irregularities in the restorations were not required.

**FIGURE 1:**
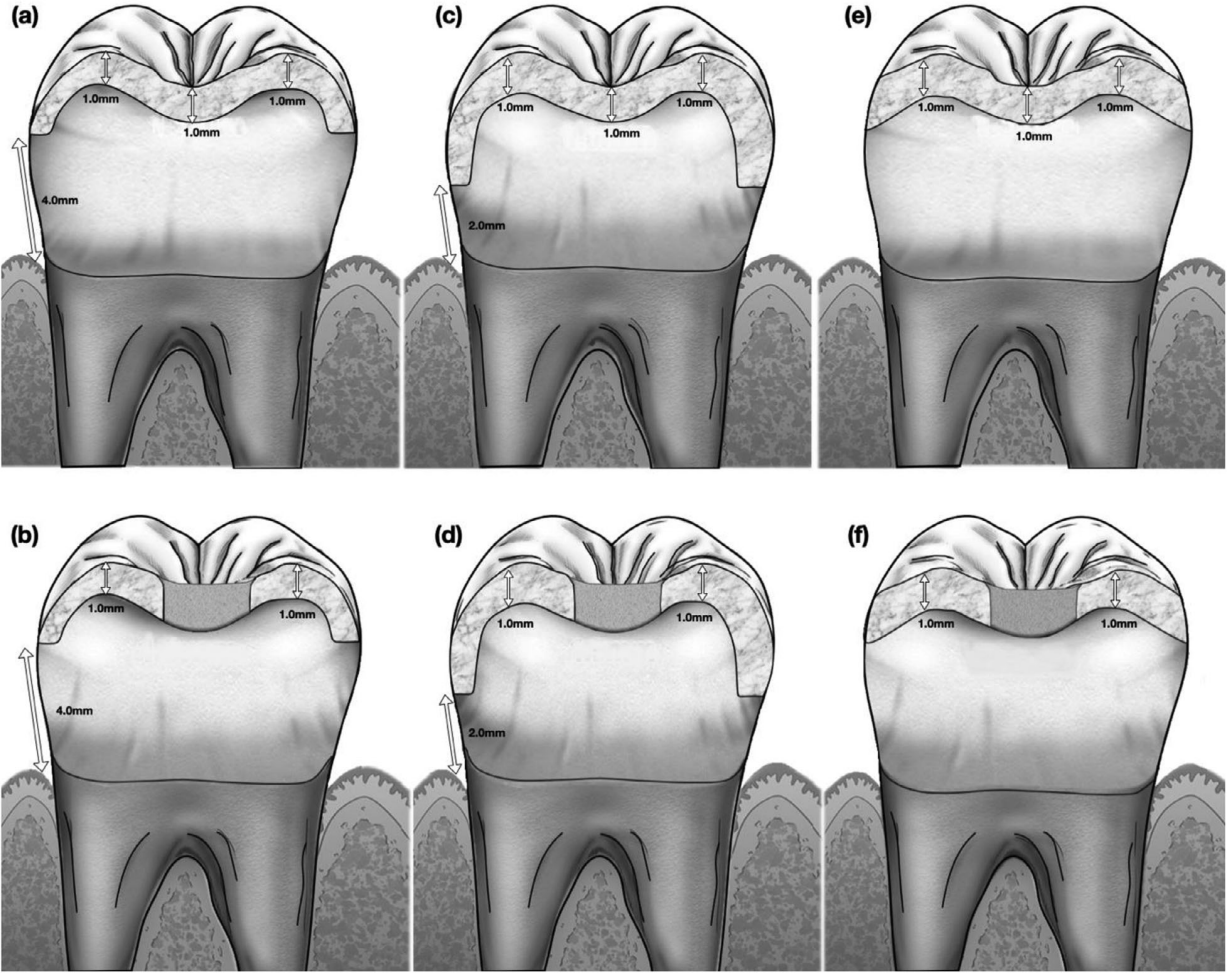
Cross‐sectional illustration of different types of overlay restorations: (a) with margin located 4 mm coronal to the gingival level without endodontic access (Group 1); (b) with margin located 4 mm coronal to the gingival level with endodontic access (Group 2); (c) with margin located 2 mm coronal to the gingival level (Group 3); (d) with margin located 2 mm coronal to the gingival level and with endodontic access (Group 4); (e) without margin (Group 5); and (f) without margin and with endodontic access (Group 6).

Group 1 (M4) had a 4 mm supragingival finish line and no endodontic access; group 2 (M4End) had a 4 mm supragingival finish line and endodontic access. Group 3 (M2) had a chamfer finish line located 2 mm above the gingival level, group 4 (M2End) had a chamfer finish line located 2 mm coronal to the gingival level and endodontic access, group 5 (nM) had no finish line, and group 6 (nMEnd) had no finish line but with endodontic access (Figure 1). To fabricate the printed resin dies, the prepped typodont teeth were scanned with a laboratory scanner (Degree of Freedom HD, DOG Seoul, Korea) and digitally designed to match the tooth preparations, and ninety dies were printed from the resin model (Gray Model Resin, FormLab 3, Formlabs, Somerville, MA, USA).

Restorations were treated first by sandblasting with 50 µm aluminum oxide for 15 s at a distance of 10 mm and 2.5 bar pressure. The restorations were then cleaned with a cleaning paste (Ivoclean, Ivoclar Vivadent) for 20 s, rinsed with water, and universal primer (Monobond Plus, Ivoclar Vivadent) was applied for 60 s, followed by air‐drying. All the indirect restorations were adhesively cemented to the resin dies with a self‐curing luting composite with a light‐curing option (Multilink Automix, Ivoclar Vivadent) and light‐cured (Elipar 2500, 3 M, St Paul, MN, USA) for 20 s on the mesial, distal, buccal, lingual, and occlusal surfaces before being left to self‐cure for 6 min with 200 g of applied weight (200G Calibration Weight, American Weigh Scales, Cumming, GA, USA).

Endodontic access for groups 2 (M4End), 4 (M2End), and 6 (nMEnd) was performed using a specialized round diamond bur for endodontic access (6801 DC, ESX Modern Access Kit, Brasseler USA, Savannah, GA, USA) with water coolant. The restoration access was then repaired using a specialized ceramic repair system (Intraoral Repair Kit, BISCO Inc., Schaumburg, IL, USA), following the manufacturer's instructions. First, the barrier gel was placed to protect the resin die surface, then the 9.5% ceramic etchant was applied for 90 s, rinsed, and air‐dried. Next, the primer was applied for 30 s and air‐dried, then zirconia primer (Z‐Primer, Bisco) was applied for 5 s and air‐dried. Porcelain bonding resin was applied to the surface, and a flowable resin composite (Filtek Supreme Flowable, 3M Oral Care, St Paul, MN, USA) was applied and then light‐cured for 20 s. All restorations were stored in distilled water at 37°C for 24 h.

To simulate crack growth caused by differential expansion and contraction of the SCs, all restorations underwent thermocycling between 5 and 55°C for 100,000 cycles with a 30‐s dwell time. The SCs were then embedded up to 2 mm below the cementoenamel junction using self‐curing acrylic resin before being loaded to compression failure using a universal testing machine (INSTRON 5965, Bluehill 3 software, USA). Each SC was placed on a jig at a 90‐degree inclination to the tooth axis and load, and a 1.5 mm rubber sheet was inserted between the crown and indenter to simulate a food bolus and distribute the load. A compressive loading rate of 1 mm/min was applied at a load of 5 kN, and the maximum load (ML) in newtons (N) and fracture resistance at maximum load (FRML) in megapascals (MPa) were recorded. Scanning electron microscopy (SEM) images of the fractured specimens were taken using a microscope (FE‐SEM JSM 6701F, Jeol Ltd, Tokyo, Japan), and the number of cracks and their lengths were quantified at 14 and 40 magnifications. The sample size was calculated through power analysis,^22^, ^31^ which demonstrated that 11 to 40 specimens were required for each group. Therefore, 15 specimens per group were considered appropriate for the in vitro study.

### Statistical analyses

The statistical analysis was conducted using SPSS statistical software (version 27, IBM, NY, USA). The normality of the data was assessed using the Levene and Shapiro‐Wilk tests, which did not find statistical significance. A one‐way analysis of variance (ANOVA) and Tukey post‐hoc tests were performed to evaluate the data. A standard level of significance was set at alpha <0.05. An independent *t*‐test was conducted to compare the groups with and without endodontic access.

## RESULTS

### Fracture test

Table 1 displays the fracture load in newtons (N) and FRML of CAD‐CAM zirconia overlay restorations with different designs, both with and without endodontic access. One‐way ANOVA revealed a statistically significant difference between the groups with and without endodontic access in terms of fracture resistance. Tables S1 and S2 present the detailed information on pairwise comparisons. The prosthetic design and presence of endodontic access had an impact on overlay restorations. The overlays in group 3 exhibited the highest fracture resistance values and were statistically significantly higher compared to the other groups.

**TABLE 1:** Fracture load and fracture resistance at maximum load of zirconia overlays with different preparation designs with and without endodontic access.

Group	Type of restoration	Fracture load (±SD), N	Fracture resistance at maximum load (±SD), MPa
Group 1 (M4)	Overlay restoration with finish line at 4 mm from gingival margin	567.07 (58.48)^a^	22.70 (2.27)^a^
Group 2 (M4End)	Overlay restoration with 4 mm finish line and endodontic access	458.05 (65.36)^b^	19.85 (1.53)^b^
Group 3 (M2)	Overlay with finish line located 2 mm coronally to gingival margin	959.27 (109.87)^c^	28.43 (2.80)^c^
Group 4 (M2End)	Overlay with margin located 2 mm above the gingiva and with endodontic access	842.94 (135.97)^d^	25.10 (2.68)^d^
Group 5 (nM)	Occlusal veneer (no margin overlay)	543.01 (41.69)^ab^	22.18 (1.37)^ab^
Group 6 (nMEnd)	Occlusal veneer with endodontic access	502.10 (40.09)^ab^	20.48 (1.61)^ab^

*Note*: Different superscript uppercase letters indicate significant difference (*p* < 0.05) within groups in each column. Fifteen specimens per group were tested.

Abbreviations: SD, standard deviation; N, Newtons; MPa, megapascals.

See Tables S1 and S2 for detailed information on pairwise comparisons.

### Fractographic analysis

**FIGURE 2:**
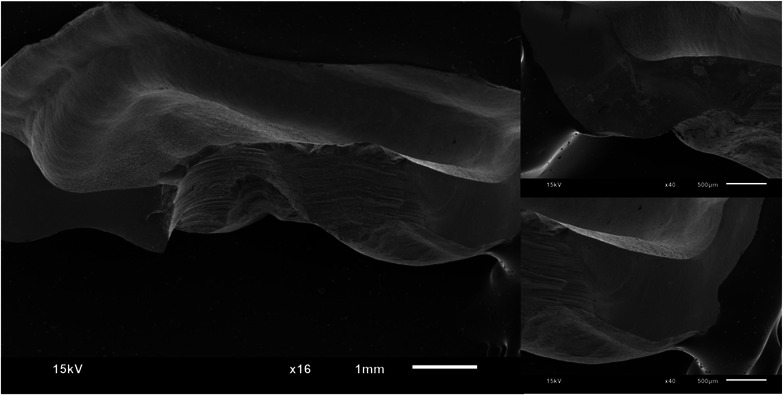
Representative scanning electron microscopy (SEM) image of group 1 overlay restoration at ×16 and ×40 magnification.

**FIGURE 3:**
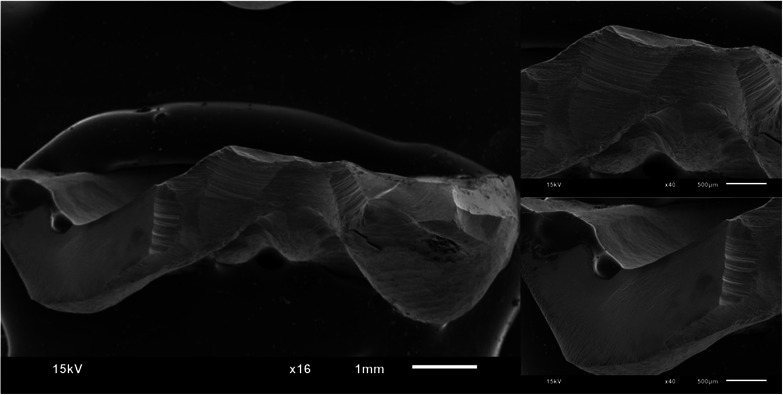
Representative scanning electron microscopy (SEM) image of group 2 overlay restoration with endodontic access at ×16 and ×40 magnification.

**FIGURE 4:**
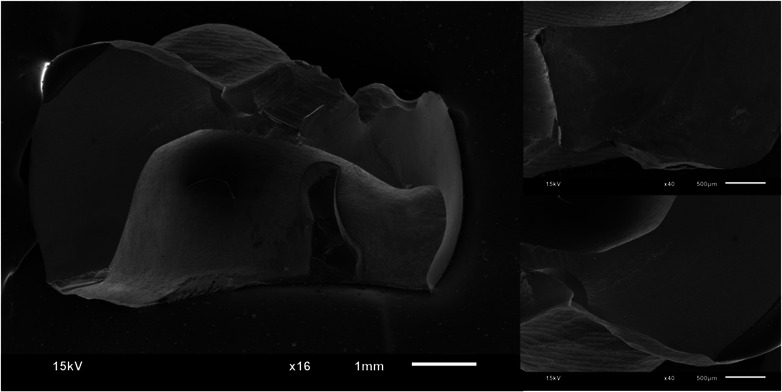
Representative scanning electron microscopy (SEM) image of group 3 overlay restoration with margin located 2 mm coronally to gingival level at ×16 and ×40 magnification.

**FIGURE 5:**
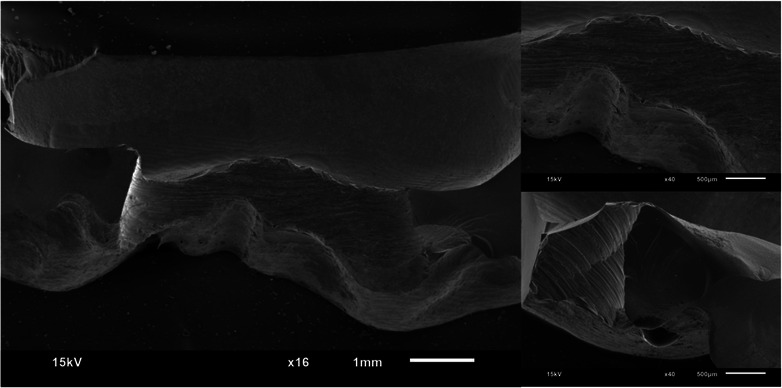
Representative scanning electron microscopy (SEM) image of group 4 overlay restoration with margin located 2 mm coronally to gingival level and with endodontic access at ×16 and ×40 magnification.

**FIGURE 6:**
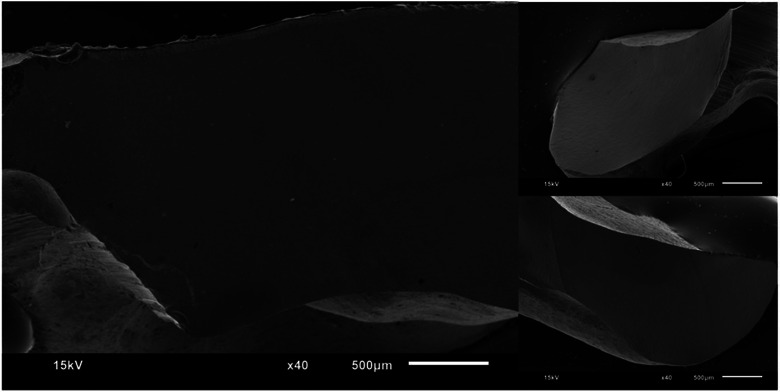
Representative scanning electron microscopy (SEM) image of group 5 overlay restoration without margin at ×16 and ×40 magnification.

**FIGURE 7:**
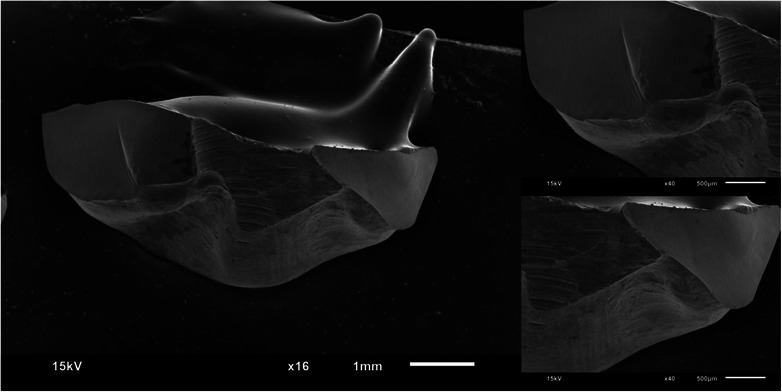
Representative scanning electron microscopy (SEM) image of group 6 overlay restoration with no margin and with endodontic access at ×16 and ×40 magnification.

Figures 2, 3, 4, 5, 6, 7 depict representative SEM images of the fractured zirconia overlay restorations with various designs, with and without endodontic access. Restorations with endodontic access exhibited a more irregular and larger quantity of crack lines compared to those without endodontic access. Additionally, smaller restorations, like those with no margin, displayed fewer and cleaner cracks than larger restorations, such as those with a chamfer margin located 2 mm above the gingival level.

## DISCUSSION

The aim of this study was to determine the fracture resistance of zirconia overlay restorations of three different designs, with and without endodontic access, for the mandibular right first molar. The first null hypothesis, which stated that the zirconia overlay of three different designs does not exhibit a difference in fracture resistance, was rejected. Group 3 displayed the highest fracture resistance values compared to all groups (*p* < 0.05), followed by group 4. The lowest fracture resistance was observed in group 2, however, it was not statically significantly lower than both groups without margin (with and without endodontic access).

The findings align with previous studies that used different materials. For example, one study compared the fracture resistance of CAD‐CAM lithium disilicate SCs, overlay restorations with a margin located 2 mm supragingivally, and overlay restorations with a margin located 4 mm supragingivally. The study found that overlays with a margin located 2 mm coronally to the gingival margin (813 N) had almost twice the fracture resistance compared to overlays with a margin located 4 mm supragingivally (436 N).^32^ Another study evaluated the fracture resistance of partial coverage restorations from two chairside CAD‐CAM leucite‐reinforced ceramic brands (IPS Empress CAD; Ivoclar Vivadent, an. Rosetta BM; Bio Hass).^33^ Both brands showed that restorations with a  4 mm margin at the gingival level had higher fracture resistance than those with a 2 mm supragingival margin.^33^

Additionally, overlays with varying preparation designs, both with and without endodontic access, exhibited distinct fracture resistance patterns. Specifically, group 4 displayed significantly higher fracture resistance values compared to group 2 and group 6. This outcome rejects the second null hypothesis, indicating that the preparation designs, presenting endodontic access, influenced the fracture resistance outcomes.

While 5Y‐PSZ exhibits lower flexural strength than 3Y‐TZP, thus, was originally intended for the esthetic zone. However, recent studies have reported that its fracture resistance is suitable for posterior full‐coverage monolithic restorations.^34^, ^35^, ^36^ Additionally, mandibular posterior occlusal surfaces might demand high aesthetics when visible (i.e., when talking or laughing) in the person. Thus, it is important to assess its suitability for load‐bearing posterior partial‐coverage single‐unit restorations, such as onlays. Partial coverage restorations have gained popularity as a treatment option in recent years, with promising results demonstrated in clinical studies. For instance, a controlled clinical trial evaluated ceramic partial coverage posterior restorations on 22 patients over 5.5 years and reported an 88.8% survival rate.^37^ A recent systematic review also found high survival rates of 91%–100% at 2–5 years and 71%–98.5% for more than 5 years for partial ceramic restorations.^38^ The review concluded that partial ceramic restorations are a reliable option for posterior teeth regardless of the follow‐up duration.^38^

The results of the current study showed that the fracture resistance of overlay restorations was significantly decreased by endodontic access, regardless of the design type. These findings are consistent with prior research. A recent in‐vitro study evaluated full‐coverage zirconia SCs with varying thicknesses (0.5, 1.0, 1.5, and 2.0 mm) for mandibular second molars, with and without endodontic access. The study found that all restorations exhibited reduced fracture resistance, with 41% less resistance for SCs with endodontic access and 0.5 mm thickness, 47% less resistance for SCs of 1.0 mm thickness, 13% less resistance for SCs of 1.5 mm thickness, and 7% less resistance for SCs of 2.0 mm thickness.^39^ Another study evaluated the fracture resistance of pressed and CAD‐CAM lithium disilicate full‐coverage SCs, with and without endodontic access. The results indicated that pressed (1901 N) and CAD‐CAM (1429 N) SCs without access presented higher fracture resistance than pressed (1573 N) and CAD‐CAM (1297 N) with endodontic access.^40^ Regarding restorations with or without a finish margin, a finite element study evaluated the stress resistance of overlays with and without a finish line for a molar. The study evaluated the stress resistance of overlays made of lithium disilicate and resin composite SCs on enamel and dentin tooth structures. The results showed that restorations without a finish line exhibited lower stress resistance for both materials (LD: 3,581 MPa; RC: 3,519 MPa) compared to those with a finish line (LD: 4,297 MPa; RC: 4,133).^41^ Moreover, the study found that restorations without a finish line displayed lower resistance than those with a finish chamfer.

Thermocycling is a widely accepted method to simulate the aging of SCs by exposing materials to fatigue. It involves abrupt temperature changes by submerging specimens in baths that stimulate anisotropy due to thermal expansion and conductivity, resulting in stress.^42^ Typically, 10,000 thermocycles represent 1 year of clinical function, as 20 to 50 cycles are equivalent to a single day.^43^ Most existing studies have simulated 5000 to 10,000 cycles before fracturing the restoration.^24^, ^44^, ^45^ Some studies have also reported providing 50,000 or more cycles.^46^  In the current study, 100,000 cycles were performed to mimic 10 years of clinical service before fracture evaluation because this period is more realistic in clinical scenarios for zirconia ceramic.

The fracture resistance values in this study, ranging from 567.07 N for group 1 (M4) to 959 N for group 3 (M2), appear to be clinically acceptable. Research indicates that occlusal forces during chewing and biting typically reach around 100 N, with a maximal bite force in habitual occlusion of up to 320 N.^47^ Additionally, electromyography studies have demonstrated human chewing forces of 364 N for almonds and up to 239 N for chewing gum.^48^ These findings are significantly lower than the fracture resistance values, suggesting that partial restorations with and without endodontic access can safely withstand occlusal forces.

The design of the restorations has followed previous studies in which they also evaluated fractured resistance of partial restorations but without endodontic access and with different types of materials.^32^, ^49^ Given this treatment's popularity and acceptable results, the current study evaluated zirconia partial coverage restorations with different designs for mandibular first molars. Various designs for partial restorations covering the entire occlusal surface, such as following the occlusal anatomy, flat occlusal surface, with finish margin located at different cervical‐occlusal heights, or even without finish margin, have been described in case reports.^11^, ^32^, ^49^ Moreover, endodontic access through ceramic restorations has become more common for practitioners.^25^, ^50^ Thus, the current study evaluated three different designs for overlay restorations, including those with a chamfer margin located at 4 mm and 2 mm, supragingivally, and those without a chamfer margin. In terms of the clinical implications of this study, clinicians should consider the location of the finish line when designing zirconia overlay restorations, as those placed 2 mm above the gingival margin demonstrated superior fracture resistance. Furthermore, when zirconia overlays undergo endodontic access it can decrease their overall longevity in clinical practice.

To the best of the authors’ knowledge, there is a lack of studies that have conducted fractographic analysis using SEM for posterior partial restorations, both with and without endodontic access. Within the scope of this research, restorations without endodontic access exhibited cleaner and more regular crack lines, while those with endodontic cavities displayed a higher occurrence of irregular crack patterns. A prior study examining teeth with and without endodontic access reported a higher rate of pulp floor fractures in cases with endodontic access compared to those without.^51^ The present study aligns with this observation, as restorations with endodontic cavities also exhibited more crack lines than those without.

This study had inherent limitations that are typical of an in vitro design. Experiments were performed on resin dies as a dentine substitute instead of natural teeth, as this material has a similar tensile strength (61 MPa) to dentin (44 to 97 MPa).^52^, ^53^ Similar approaches have been adopted in other studies.^25^, ^41^, ^50^ However, this resin is not entirely clinically realistic. Using natural teeth may provide more variables for testing, such as methods of collecting teeth without caries, preparing teeth, and storing and handling natural teeth. Another limitation of this study is the absence of a group that did not undergo thermal cycling. Thermomechanical loading can be explored in future studies to enhance clinical relevance by simulating occlusal forces and temperature fluctuations experienced by restorations in the oral environment. A control group could have provided a better comparison and assessment of the effects of thermocycling on the fracture resistance of the tested restorations. Inspections following the fabrication of restorations were omitted due to the uniform quality observed across CAD‐CAM specimens, corroborated by standard deviations found in Table 1 data. Not inspecting the restorations for flaws prior to endodontic access might be treated as a potential confounder. The endodontic access performed through the restoration mirrors a clinical scenario requiring immediate endodontic therapy after restoration placement. Future studies may include groups simulating aging before the endodontic access, enabling a comprehensive evaluation of their combined impact on overlay restoration fracture resistance. Lastly, shorter and longer fatigue cycling may have helped to better predict the performance of the restoration over the short‐ and long‐term. While the current study used 100,000 cycles to simulate 10 years of clinical service, shorter and longer cycles could have provided valuable insights into the fracture resistance of restorations in different simulated clinical scenarios. Future studies in clinical settings might be necessary to confirm the findings and improve the clinical applicability of zirconia overlay restorations.

## CONCLUSIONS

Within the limitations of the present in vitro study, the findings indicated that the zirconia overlay restorations with different preparation designs exhibited varying fracture resistance, with higher statistically significant values observed in overlays featuring a 2 mm supragingival margin compared to those with more supragingival finish lines or without a margin. Overlays of distinct preparation designs presenting endodontic access displayed significantly lower fracture resistance compared to those without endodontic access within the same design, except for the no‐margin preparation design. Furthermore, no statistically significant difference was observed between the 4‐mm supragingival overlays with and without endodontic access, compared to overlays without margins, regardless of endodontic access.

## Supporting information

Supporting Information
