# Supplementary material for: Effect of preparation design and endodontic access on fracture resistance of zirconia overlays in mandibular molars: An in vitro study
Source: J Prosthodont. 2024 May 12;34(4):412–21. doi: 10.1111/jopr.13865 (PMC11976686; doi:10.1111/jopr.13865)
Supplement: Supplementary file 1 — Supporting Information [file JOPR-34-412-s001.docx]

Supplementary Information

**SUPPLEMENTARY TABLE 1** Pairwise comparisons of the fracture load values of computer-aided design and computer-aided manufacturing (CAD–CAM) zirconia overlays

|  | Group 1 (M4) | Group 2 (M4End) | Group 3 (M2) | Group 4 (M2End) | Group 5 (nM) | Group 6 (nMEnd) |
| --- | --- | --- | --- | --- | --- | --- |
| Group 1 (M4) |  | 0.011 | < 0.001 | < 0.001 | 0.973 | 0.318 |
| Group 2 (M4End) | 0.011 |  | < 0.001 | < 0.001 | 0.088 | 0.728 |
| Group 3 (M2) | < 0.001 | < 0.001 |  | 0.005 | < 0.001 | < 0.001 |
| Group 4 (M2End) | < 0.001 | < 0.001 | 0.005 |  | < 0.001 | < 0.001 |
| Group 5 (nM) | 0.973 | 0.088 | < 0.001 | < 0.001 |  | 0.785 |
| Group 6 (nMEnd) | 0.318 | 0.728 | < 0.001 | < 0.001 | 0.785 |  |

Abbreviations: M4, with finish line 4 mm coronally to gingival margin; M2, with finish line 2 mm coronally to gingival margin; nM, without margin (occlusal veneer); End, with occlusal endodontic access.

**SUPPLEMENTARY TABLE 2** Pairwise comparisons of the fracture resistance at maximum load values of computer-aided design and computer-aided manufacturing (CAD–CAM) zirconia overlays

|  | Group 1 (M4) | Group 2 (M4End) | Group 3 (M2) | Group 4 (M2End) | Group 5 (nM) | Group 6 (nMEnd) |
| --- | --- | --- | --- | --- | --- | --- |
| Group 1 (M4) |  | 0.008 | < 0.001 | 0.040 | 0.988 | 0.075 |
| Group 2 (M4End) | 0.008 |  | < 0.001 | < 0.001 | 0.052 | 0.970 |
| Group 3 (M2) | < 0.001 | < 0.001 |  | 0.001 | < 0.001 | < 0.001 |
| Group 4 (M2End) | 0.040 | < 0.001 | 0.001 |  | 0.006 | < 0.001 |
| Group 5 (nM) | 0.988 | 0.052 | < 0.001 | 0.006 |  | 0.284 |
| Group 6 (nMEnd) | 0.075 | 0.970 | < 0.001 | < 0.001 | 0.284 |  |

Abbreviations: M4, with finish line 4 mm coronally to gingival margin; M2, with finish line 2 mm coronally to gingival margin; nM, without margin (occlusal veneer); End, with occlusal endodontic access.
